# Mouse parotid salivary gland organoids for the in vitro study of stem cell radiation response

**DOI:** 10.1111/odi.13475

**Published:** 2020-06-29

**Authors:** Paola Serrano Martinez, Davide Cinat, Peter van Luijk, Mirjam Baanstra, Gerald de Haan, Sarah Pringle, Robert P. Coppes

**Affiliations:** ^1^ Department of Biomedical Sciences of Cells and Systems University Medical Center Groningen University of Groningen Groningen The Netherlands; ^2^ Department of Radiation Oncology University Medical Center Groningen University of Groningen Groningen The Netherlands; ^3^ European Research Institute for the Biology of Ageing University Medical Center Groningen University of Groningen Groningen The Netherlands; ^4^ Department of Rheumatology and Clinical Immunology University Medical Center Groningen University of Groningen Groningen The Netherlands

**Keywords:** mouse parotid gland, organoids, parotid gland stem cells, radiosensitivity

## Abstract

**Objective:**

Hyposalivation‐related xerostomia is an irreversible, untreatable, and frequent condition after radiotherapy for head and neck cancer. Stem cell therapy is an attractive option of treatment, but demands knowledge of stem cell functioning. Therefore, we aimed to develop a murine parotid gland organoid model to explore radiation response of stem cells in vitro.

**Materials and Methods:**

Single cells derived from murine parotid gland organoids were passaged in Matrigel with defined medium to assess self‐renewal and differentiation potential. Single cells were irradiated and plated in a 3D clonogenic stem cell survival assay to assess submandibular and parotid gland radiation response.

**Results:**

Single cells derived from parotid gland organoids were able to extensively self‐renew and differentiate into all major tissue cell types, indicating the presence of potential stem cells. FACS selection for known salivary gland stem cell markers CD24/CD29 did not further enrich for stem cells. The parotid gland organoid‐derived stem cells displayed radiation dose–response curves similar to the submandibular gland.

**Conclusions:**

Murine parotid gland organoids harbor stem cells with long‐term expansion and differentiation potential. This model is useful for mechanistic studies of stem cell radiation response and suggests similar radiosensitivity for the parotid and submandibular gland organoids.

## 1 INTRODUCTION

Hyposalivation‐related xerostomia is a major problem for patients treated with radiotherapy for head and neck cancer. The damage induced by the co‐irradiation of salivary glands (SGs) results in severe hyposalivation‐related side effects, such as oral dryness, difficulty with normal oral functions, loss of taste, ulcerations, and an increased risk of developing dental caries and oral infections. Consequently, the quality of life of these patients is drastically diminished (Langendijk et al., 2008; Pringle, Van Os, & Coppes, 2013). Strategies to avoid harm to the SGs include the use of protective drugs (Bohuslavizki et al., 1998; Brizel et al., 2000; Burlage et al., 2008; LeVeque et al., 1993), relocation of the submandibular gland by surgery (Jha, Seikaly, McGaw, & Coulter, 2000), and SG‐sparing radiotherapy techniques (Eisbruch, 2009; Feng et al., 2010; Kam et al., 2007; Murdoch‐Kinch, Kim, Vineberg, Ship, & Eisbruch, 2008; Nutting et al., 2011; Vissink, van Luijk, Langendijk, & Coppes, 2015). However, there is no satisfactory therapy to treat radiation‐induced SG damage.

Saliva is mostly produced by the major salivary glands, namely the parotid, submandibular, and sublingual glands. The submandibular glands (SMGs) contribute to 65% of the unstimulated saliva, 20% is produced by the parotid gland (PG) and 7%–8% by the sublingual glands (SLG) (Humphrey & Williamson, 2001). Upon stimulation, the PG generates up to 50% of secreted saliva (Dirix & Nuyts, 2010). The PG is thus the major source of stimulated saliva (Humphrey & Williamson, 2001). The degree of salivary gland hypofunction is directly related to the amount of radiation dose delivered to the glands. Preclinically, PG and SMG have been shown to differ in sensitivity (Coppes, Vissink, & Konings, 2002). However, there seems to be a discrepancy in the field of the threshold of the radiation dose that can be delivered to the PG or the SMG for expecting recovery after irradiation (Li, Taylor, Ten Haken, & Eisbruch, 2007; Murdoch‐Kinch et al., 2008). In the clinic, dosimetric sparing of the PGs is possible, allowing the reduction of the probability of severe xerostomia development in these patients (Deasy et al., 2010; Dijkema et al., 2010; Dirix & Nuyts, 2010; Jellema, Doornaert, Slotman, Leemans, & Langendijk, 2005; Kam et al., 2007; Nutting et al., 2011). Nevertheless, not all patients can benefit from SG‐sparing radiotherapy, owing to its dependency on the stage of disease and tumor location (Chen et al., 2017). After radiotherapy, it has been reported that a certain degree of PG function may recover (Braam et al., 2005; Braam, Roesink, Raaijmakers, & Terhaard, 2007). This improvement accounts however for 32% of salivary flow rate, with 41% of patients still suffering of moderate or severe xerostomia 5 years postirradiation. Patients receiving higher doses (>46 Gy of fractionated radiation) will eventually suffer from irreversible parotid dysfunction (Braam et al., 2005). Therefore, there is a need for development of new strategies for the treatment of radiation‐induced xerostomia and increased focus on the PG specifically.

In regenerative medicine, stem cell therapy is an attractive option for the long‐term treatment of hyposalivation‐related xerostomia induced by irradiation. The partial functional recovery of the PG after certain threshold of irradiation suggests the existence of stem or progenitor cells (indistinguishable from each other in this context, from here forward termed “stem cells”) able to proliferate and differentiate, allowing tissue regeneration upon damage. We have reported the isolation of cells from the murine (Maimets et al., 2016; Nanduri et al., 2014) and the human SMG (Pringle et al., 2016) that have shown in vitro self‐renewal and differentiation potential, both stem cell characteristics. Moreover, the derived cells from these organoids exhibit in vivo engraftment and the capacity to restore saliva production in irradiated mice (Maimets et al., 2016) further indicating their stem cell characteristic. In the case of the PG, isolation of stem cells derived from human parotid gland (HPG) samples has been described (Pringle et al., 2019). These parotid stem/progenitor cells showed ability to self‐renew and to generate mature amylase expressing organoids. However, extensive research is still warranted, in order to define the nature of the specific human parotid stem cells and their in vivo potential to restore PG function. Difficulty in obtaining HPG tissue for such studies places the use of murine parotid stem cells as viable alternative option, which might provide the basic knowledge required to optimize human organoid cultures and enable irradiation studies. To this end, adult rodent parotid cells have been isolated and used to establish PG salisphere cultures (Chibly et al., 2018; Leigh, Nelson, Mellas, McCall, & Baker, 2017; McCall et al., 2013; Nguyen, Dawson, Zhang, Harris, & Limesand, 2018; van Luijk et al., 2015). Specifically, clusters of parotid‐derived cells were able to generate primary salispheres, which after dissociation lead to secondary spheres expressing amylase (Chibly et al., 2018). However, the ability of the cells derived from these cultures to expand extensively and to differentiate into all the adult parotid cell lineages, such as achieved with SMG organoids, has not been demonstrated. The clinical relevance of murine models for the prediction of PG function has been established, since regional radiation treatments predicted functional outcomes similarly for rats and humans (van Luijk et al., 2015). Therefore, the aim of this study is to develop a murine PG organoid model and to explore the radiation response of its stem cells in vitro. Using a 3D organoid culture model, we report that the adult murine PG harbors stem cells with extensive self‐renewal and multipotency. Moreover, our model can be used for the assessment of the in vitro parotid stem cell response to radiotherapy, in order to further develop stem cell therapies and to optimize regenerative potential of salivary gland tissue affected by the treatment of head and neck cancer. These approaches may lead to an improvement in the quality of life for head and neck cancer patients.

## 2 MATERIALS AND METHODS

### 2.1 Mice

Eight‐ to 10‐week‐old female C57BL/6 mice were purchased from Envigo and were bred in the Central Animal Facility of University Medical Center Groningen (Groningen, The Netherlands). The mice were maintained under conventional conditions and fed ad libitum with food pellets (RMH‐B; Hope Farms B.V.) and water. All experiments were approved by the Ethical Committee on animal testing of the University of Groningen.

### 2.2 Immunofluorescence

Analysis of the expression of SG ductal and acinar markers was assessed by immunofluorescence. Extirpated PGs were fixed in 4% formaldehyde (24 hrs, room temperature (RT)). After dehydration, the tissue was paraffin‐embedded and sectioned at 4 μm thickness. For the case of organoids, Matrigel was dissolved by incubation with Dispase enzyme (1 mg/ml for 30 min to 1 hr at 37°C; Sigma). Released organoids were washed with PBS/0.2% BSA and centrifuged at 400 *g* for 5 min. The resulting pellet of organoids was dissolved in 4% paraformaldehyde for fixation (15 min, RT) and washed with 1× PBS. Next, the organoids were embedded in HistoGel (Richard‐Allan Scientific/Thermo scientific) and the gel containing the organoids was subjected to dehydration, followed by embedding in paraffin and sectioning (4 μm thickness). The tissue or the organoid sections were dewaxed, boiled for 8 min in preheated 10 mM citric acid (Sigma‐Aldrich)/10 mM sodium citrate (Sigma‐Aldrich) retrieval buffer pH 6.0, containing 0.1% Tween 20. After washing thoroughly, the following primary antibodies were used: cytokeratin 14 (CK14, 1:100, Abcam, ab175549), cytokeratin 8 (CK8, 1:50, Hybridoma Bank, TROMA‐I), aquaporin 5 (AQP5, 1:400, Alomone Labs, AQP‐005), and alpha‐amylase 1A (Amy, 1:100, Sigma‐Aldrich, SAB4200673). For fluorescence microscopy, Alexa Fluor 594 donkey anti‐rat (Thermo Fischer Scientific, A‐21209), Alexa Fluor 488 goat anti‐mouse (Thermo Fischer Scientific, A11001), or Alexa Fluor 588 goat anti‐rabbit (Thermo Fischer Scientific, A11008) conjugates at 1:1,000 dilution were used as secondary antibodies. Nuclear staining was performed with DAPI (Sigma‐Aldrich). Images were acquired with Leica DM6 B microscope using LAS X software. The analysis of fluorescent intensity was performed using ImageJ software (NIH Image). Three representative organoids for each marker were imaged at 40× magnification. Results were presented as the mean percentage of positively stained area, quantified from 3 organoids.

### 2.3 Primary culture

Primary culture was used for the elimination of cell debris. Dissected PGs were collected in Hank's balanced salt solution (HBSS, Gibco) containing 1% bovine serum albumin (BSA; Gibco). PGs were mechanically and enzymatically dissociated, using the gentleMACS Dissociator (Miltenyi Biotec, Bergisch Gladbach, Germany) and 2 ml of HBSS/1%BSA containing 0.063 mg/ml collagenase type II (Gibco), 0.5 mg/ml hyaluronidase (Sigma), and 50 mM calcium chloride, for one period of 15 min at 37°C, following by another mechanical dissociation in the gentleMACS. Digested tissue was collected by centrifugation and washed two times in HBSS/1% BSA solution. After every wash, the cellular suspension was collected again by centrifugation and the resulting pellet was resuspended in 1 ml of minimal medium [MM: contains DMEM/F12 (Life Technologies) medium, 1 × Pen/Strep antibiotics (Invitrogen), Glutamax (Invitrogen), 20 ng/ml epidermal growth factor (EGF; Sigma), 20ng/ml fibroblast growth factor‐2 (FGF‐2; Sigma), N2 (Gibco), 10 μg/ml insulin (Sigma), and 1 μM dexamethasone (Sigma)] and plated in 1 well of a 12‐well tissue culture plates.

### 2.4 Self‐renewal assay

Self‐renewal assay was performed in order to determine the capacity of PG stem cells to expand in vitro. After 1 day of primary culture, the cellular clumps were dissociated into a single cell suspension using 0.05% trypsin‐EDTA (Invitrogen). The cell number was calculated, and 10,000 cells were plated in 75 μl gel/well [25 μl cell suspension + 50 μl volume of Matrigel (BD Biosciences)] and deposited in the center of 12‐well tissue culture plates. After polymerization of Matrigel for 20 min at 37°C, 1ml of the corresponding medium was added gently on top of the gels and incubated for 7 days at 37°C. The added medium was EM [MM + Rho‐inhibitor, Y‐ 27632 (Sigma‐Aldrich)] or WRY (10% DMEM/F12, 1× Pen/Strep antibiotics, Glutamax, 20 ng/ml EGF, 20ng/ml FGF‐2, N2, 10 μg/ml insulin, 1μM dexamethasone and 10 μg/ml Y‐27632 10% R‐Spondin and 50% Wnt3a both derived from a producing cell line). To assess long‐term self‐renewal ability, the secondary organoids were passaged every 7 days. One week after seeding, Matrigel was dissolved by incubation with Dispase enzyme (1 mg/ml for 30 min to 1 hr at 37°C), organoids having a diameter greater than 50 μm were counted per well (Pringle et al., 2019) and percentage of organoid forming efficiency was calculated per condition. Released organoids were washed with PBS/0.2% BSA and centrifuged at 400 *g* for 5 min. The resulting pellet was processed to a single cell suspension using 0.05% trypsin/EDTA and passed through 40‐μm filter to filter out clumps. Single cells were counted and seeded (10,000 cells/well) in Matrigel. This procedure was performed at the end of every passage, and population doubling was determined. Organoid forming efficiency and population doubling were calculated as previously described (Maimets et al., 2016; Nagle et al., 2016; Pringle et al., 2019).

### 2.5 Differentiation assay

To asses differentiation capacity of PG stem cells, differentiation assays were performed. At the end of a passage in self‐renewal, medium on top of gels was removed and the Matrigel was dissolved using cold 1× PBS to release organoids from the gel. The organoids were collected and pelleted by centrifugation. Pelleted organoids were resuspended into 10× DMEM, Collagen I (Rat Tail, Corning, 354236), 1 M NaOH and Matrigel GFR (BD Biosciences) (40 collagen I:60 Matrigel GFR) mixing carefully. Next, the mix was plated immediately as 100 µl/well into a flat bottomed 96‐well plate (precoated with 40 μl of 50 DMEM/F12: 50 Matrigel) and allowed to solidify for 20 min at 37°C. After solidification, 150 μl of MM with 10% fetal calf serum (FCS), 1 μM DAPT (Sigma), and 50 ng/ml HGF (Peprotech/tebu‐bio) was added on top of each gel and incubated at 37°C. Medium was refreshed every 4th day. After 15 days in differentiation assay, the medium on top of the gels was removed, and the gels were collected into Eppendorfs containing 0.5 mg/ml Collagenase Type I and 0.5 mg/ml Dispase in DMEM/F12. Then, the Eppendorfs were placed in a shaking water bath at 37°C.

### 2.6 Flow cytometry analysis (FACS)

FACS was used for the selection of specific cell populations based on CD24/CD29 expression. At the end of the primary culture, the SMG salispheres or the PG cell clusters were collected, washed, and dissociated into single cells using 0.05% trypsin‐EDTA (Gibco, Invitrogen). Next, the cellular suspension was filtered through a 40‐μm filter. Cell pellets were incubated with anti‐mouse CD24‐FITC (BD Pharmingen, 553261) and CD29‐APC (BioLegend, 102215) antibodies for 15 min at RT. After washing thoroughly, the cells were suspended in a solution containing DAPI (1 mg/ml; Sigma‐Aldrich), MgSO_4_ (10 mM; Sigma‐Aldrich), and DNase I (50 µg/ml; Sigma‐Aldrich). For the sorting procedure, a Sony SH800S cell sorter was used. Pulse‐width gating excluded cell doublets, while dead cells were excluded by gating on DAPI‐negative cells. Positive gating was based on the comparison of non‐stained (Blank) and single antibody‐stained samples (CD29 single staining, CD24 single staining). Live cells (DAPI‐negative cells, non‐stained for CD24/CD29) were sorted as the control population. Sorted cells were embedded in Basement Membrane Matrigel (BD Biosciences) and seeded to and adjusted density of 20,000 cells per well, in a 12‐well tissue culture plates as described in the self‐renewal assay. Cells were cultured in WRY medium and after 10 days of growth, the self‐renewal potential of the different CD24/CD29 populations was compared with live cells.

### 2.7 Irradiation treatment

Irradiation assays were performed as previously described by our group (Nagle et al., 2016). Irradiation was realized using a 137Ce source (IBL 637 Cesium‐137 γ‐ray machine) with a dose rate of 0.59 Gy/min. To determine the sensitivity of the stem cells to irradiation, a 3D survival assay was performed. Cells were seeded as single cells in Matrigel as described previously, 2 hr before irradiation with 0–8 Gy. PG and SMG stem cells were seeded at a density of 2 × 10^4^ cells per well for the doses of 0–2 Gy or 6 × 10^4^ cells per well for the 4–8 Gy doses. At 1 week after irradiation, the organoids were counted. Surviving fractions were calculated as described by Nagle et al. (2016).

### 2.8 Data analysis

All values are represented as mean ± standard deviation (*SD*). Numbers for tested groups equal to 3 or more. Student's *t* test was used for testing statistical significance, and associations among variables were assessed by the Pearson correlation coefficient (*r*), using GraphPad Prism 6 software. If error bars are not visible, they were smaller than the data labels.

## 3 RESULTS

**FIGURE 1:**
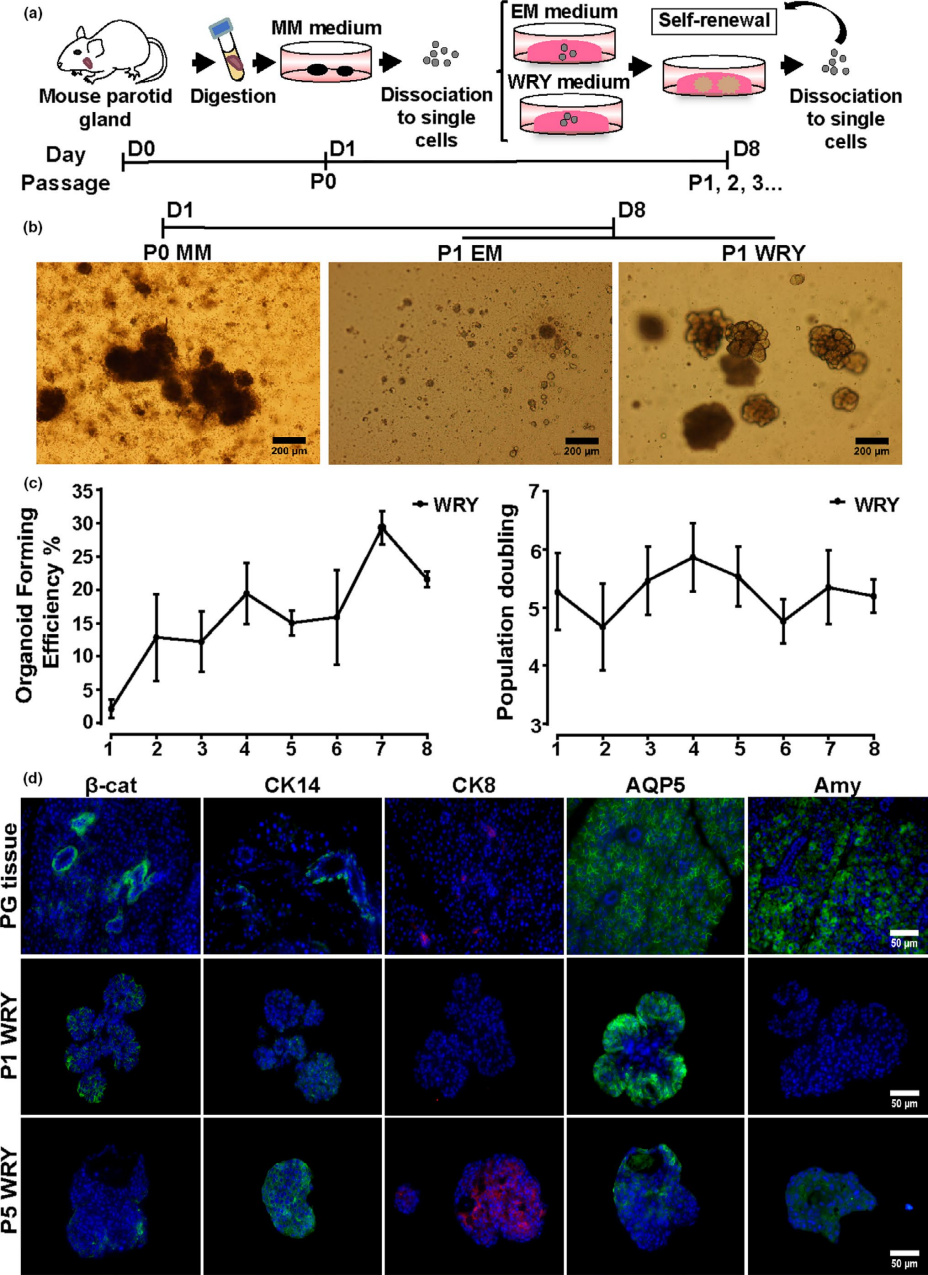
Long‐term expansion of multipotent PG stem cells. (a) Schematic representation of the isolation, primary culture (P0), and self‐renewal assay of murine PG stem cells. Minimal medium (MM). (b) Representative images of the resulting growth after primary culture or passage 1 (P1) in enriched medium (EM) or WRY (EM + Y‐drug + Wnt and R‐Spondin) medium. (c) Organoid forming efficiency and population doubling of the self‐renewal of PG stem cells in WRY medium. Error bars represent *SD*. Scale bars 200 μm. *N* = 3 biological repeats. (d) Immunofluorescence images for the Wnt signaling, and the salivary gland ductal and acinar marker expression in murine PG tissue and organoids derived after the end of P1 and P5. Beta‐catenin (β‐cat, green), cytokeratin 14 (CK14, green), cytokeratin 8, (CK8, red), aquaporin 5 (AQP5, green), amylase (Amy, green), and DAPI (blue). Scale bars 50 μm

**FIGURE 2:**
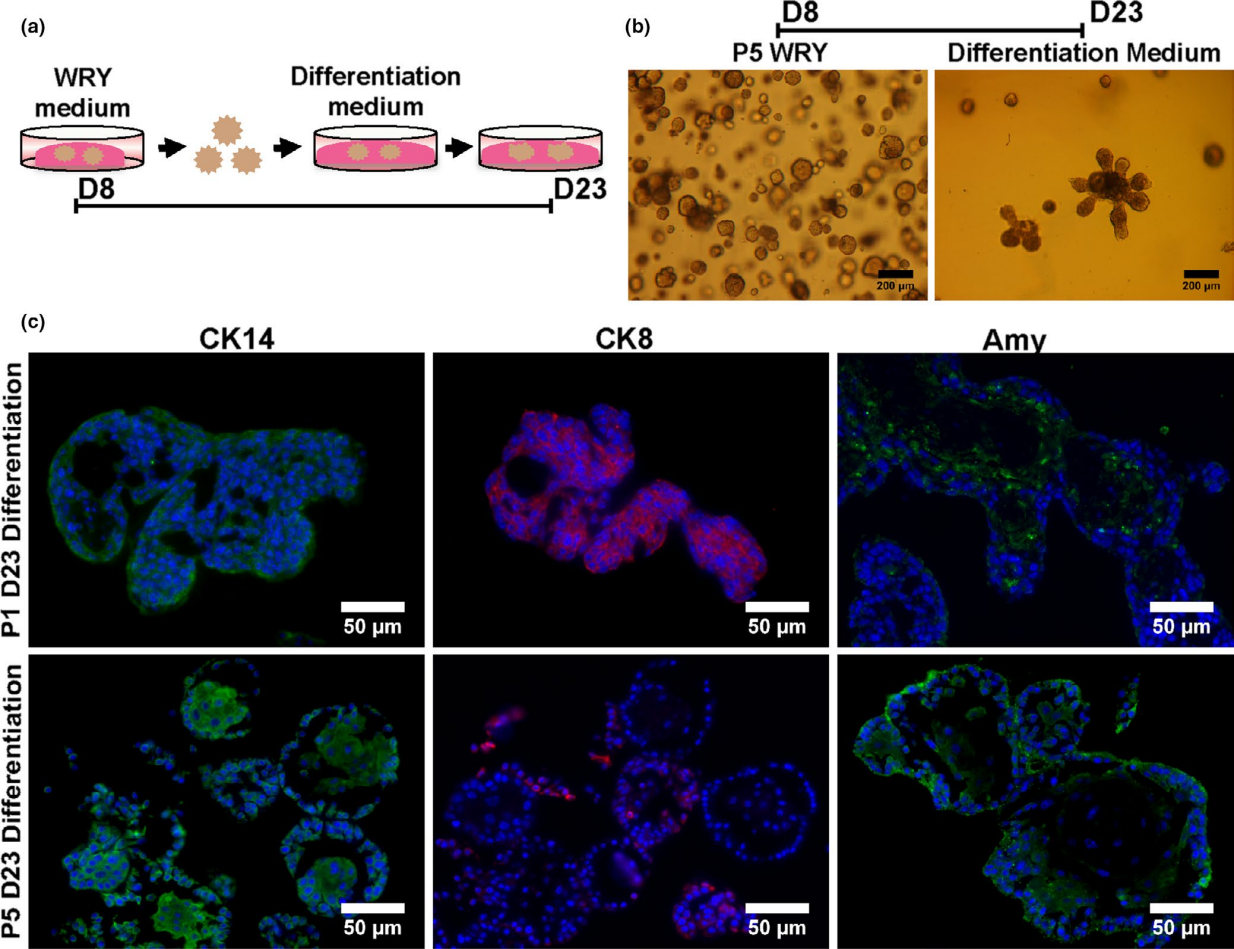
Differentiation potential of PG stem cells. (a) Diagrammatic representation of the differentiation assay in Matrigel/collagen of the derived organoids from different passages of the self‐renewal assay. (b) Representative images of the organoids derived at the end of P5 in WRY and their further morphological changes after 15 days in differentiation medium. Scale bars 200 μm. (c) Immunofluorescence images of paraffin sections of 23‐day (D23)‐old mature organoids for the analysis of expression of salivary gland ductal and acinar markers. Cytokeratin 14 (CK14, green), cytokeratin 8, (CK8, red), amylase (Amy, green), and DAPI (blue). Scale bars 50 μm

In order to assess the stemness of cells derived from murine PGs, we modified our in vitro mouse SMG organoid culture protocol (Maimets et al., 2016; Nanduri et al., 2014). Mouse PGs were excised, and the cells were separated through mechanical and enzymatic digestion. The resulted clumps of 2–3 cells were put into primary culture or Passage 0 (P0) in minimal medium (MM) for 1 day to remove the debris. Next, the remaining clusters of cells were dissociated into single cells using trypsin and seeded in Matrigel with a density of 10,000 cells per well and cultured in different medium (Figure 1a). After a period of 7 days organoids were formed, the Matrigel was digested using Dispase, and the organoids were dissociated into single cells and re‐plated in Matrigel (Passage 1, P1) (Maimets et al., 2016; Nanduri et al., 2014). To assess long‐term self‐renewal potential, this process was repeated after 7 days at the end of each passage (P2, 3, etc.). Interestingly, at P1 only cells cultured using WRY medium formed organoids (Figure 1b), but not cells cultured in EM indicating that for the development of murine PG‐derived organoids Wnt addition is essential, in contrast to SMG organoids (Maimets et al., 2016). Subsequent passaging and culturing of PG organoid‐derived cells under Wnt activation conditions resulted in self‐renewal and expansion for 2 months and longer (Figure 1c), indicative of the potential presence of stem cells. Wnt signaling potential was confirmed using immunofluorescence detection of β‐catenin (Figure 1d), being prominent in the ductal compartment and to a lesser extent in the acinar cells, similarly to the SMG (Maimets et al., 2016). P1 and P5 PG organoids showed β‐catenin expression (quantified in Figure S1b) suggesting the important role of Wnt signaling during the initiation and the expansion of these organoids. Next, we wondered whether the cells that are able to self‐renew are also able to differentiate into organoids containing the major cell types that constitute the adult PG, a second requirement defining stem cells. Therefore, we analyzed the expression of known SG ductal and acinar markers of mouse PG tissue in these organoids. To show that even after extensive self‐renewal/passaging the cells were still able to differentiate, we tested PG tissue and organoids derived from P1 and P5 using immunofluorescence (Figure 1d). Indeed, P1 organoids expressed CK14, a PG basal striated ductal marker and AQP5, an acinar cell marker, but not the luminal PG ductal marker CK8 or amylase, indicating that these organoids are not fully matured yet (Figure S1). In contrast, P5 organoids expressed all these markers suggesting a further maturation upon passaging (Figure S1). To further substantiate these findings, we exchanged WRY medium for differentiation medium (Figure 2a), known to further mature SMG organoids (Nanduri et al., 2014, Figure 2b). Indeed, here CK14, CK8, and amylase were strongly expressed after both passages (Figure 2c, Figure S1a,c) indicating the potential of the stem cells isolated from mouse PG and cultured within organoids is not only for self‐renewal but also for long‐term expansion and differentiation into the major cell types of the PG.

**FIGURE 3:**
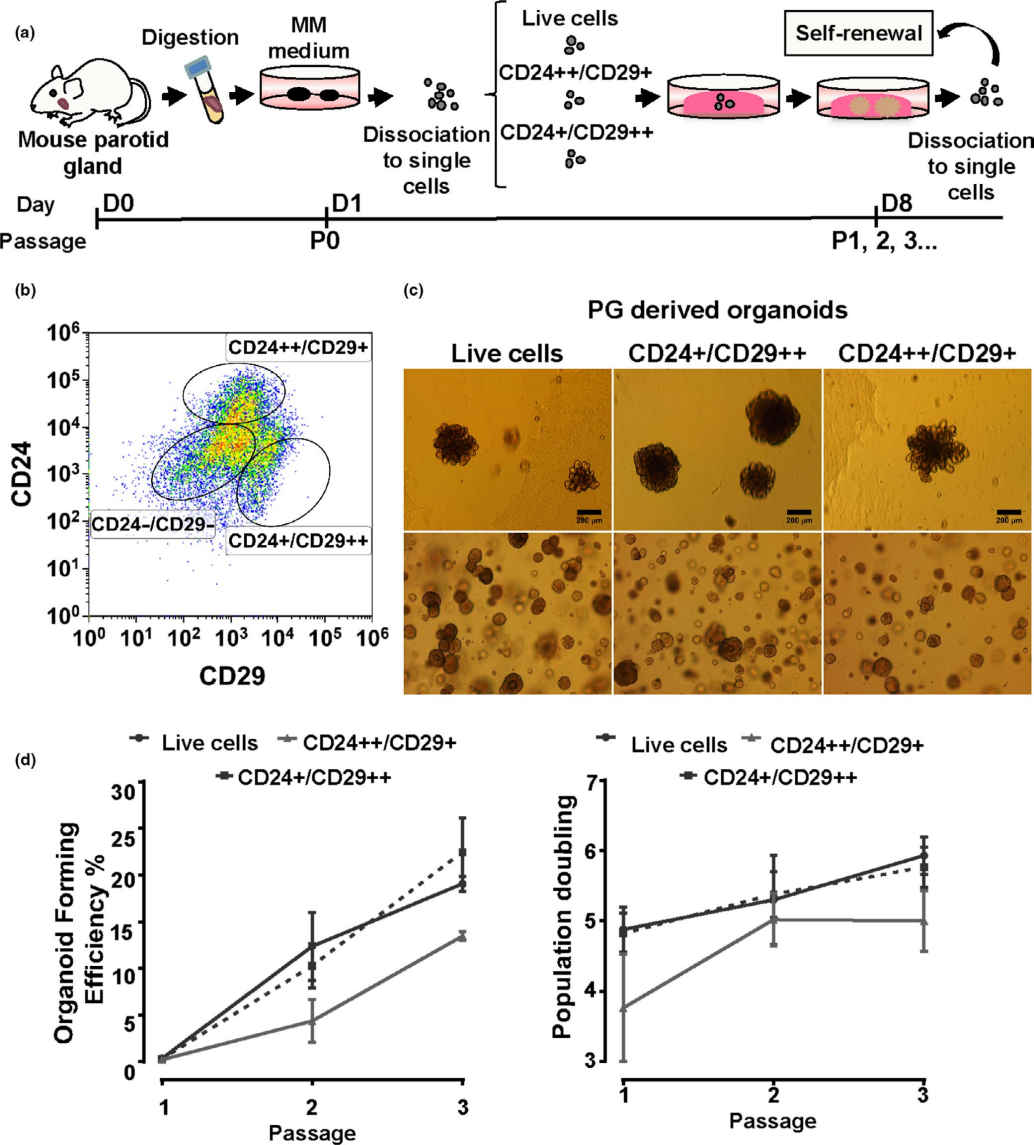
Self‐renewal potential of CD24/CD29 subpopulations of the murine PG. (a) Strategy for the determination of the self‐renewal capacity of different CD24/CD29 cell populations derived from PG primary cultures. (b) Representative FACS gating strategy for the isolation and further expansion of the CD24^++^/CD29^+^ and CD24^+^/CD29^++^ PG expressing cellular populations. The negative population for the expression of CD24 and CD29 is represented as CD24^−^/CD29^−^. (c) Representative images of organoid growth after P1 and P3 in WRY medium of the sorted live cells, and CD24^++^/CD29^+^ and CD24^+^/CD29^++^ subsets. (d) Organoid forming efficiency and population doubling of the self‐renewal culture in WRY of live cells, and CD24^++^/CD29^+^ and CD24^+^/CD29^++^ cellular subsets. Error bars represent *SD*. Scale bars 50 μm. *N* = 3 biological repeats

Next we characterized the cell population within the organoids further. CD24^++^/CD29^++^ cells derived from the mouse SMG have been shown to be enriched for stem cells (Nanduri et al., 2014). To test whether these markers are also present on the PG, we tested sorted cells derived from P0 for these two labels and used live FACS cells as control (DAPI‐negative cells, non‐stained for CD24/CD29) (Figure 3a). The populations of CD24 and CD29 expressing cells were similar to those found in SMG organoids (Figure 3b, Figure S2a,b). However, there was a difference in the fluorescence strength of the CD24^+^/CD29^++^ and CD24^++^/CD29^+^. For the case of the SMG, the CD24^++^/CD29^+^ population was more enriched, while the CD24^+^/CD29^++^ population was more abundant in the PG (Figure S2a,b). This difference did not have an influence in further results since the sorted cells were seeded at the same density for each population. Based on our previous work in the SMG, the CD24^++^/CD29^++^ population contains more putative stem cells according to their higher organoid forming efficiency (OFE) and population doubling (Nanduri et al., 2014). In contrast however to those of the SMG, neither PG cells selected for CD24^++^/CD29^+^ nor for CD24^+^/CD29^++^ improved OFE compared to all live cells (Figure 3c,d). Moreover, we did not observe differences in the size of the organoids in the different groups. These data indicate differences in stem cell enrichment marker expression in the PG compared to the SMG.

**FIGURE 4:**
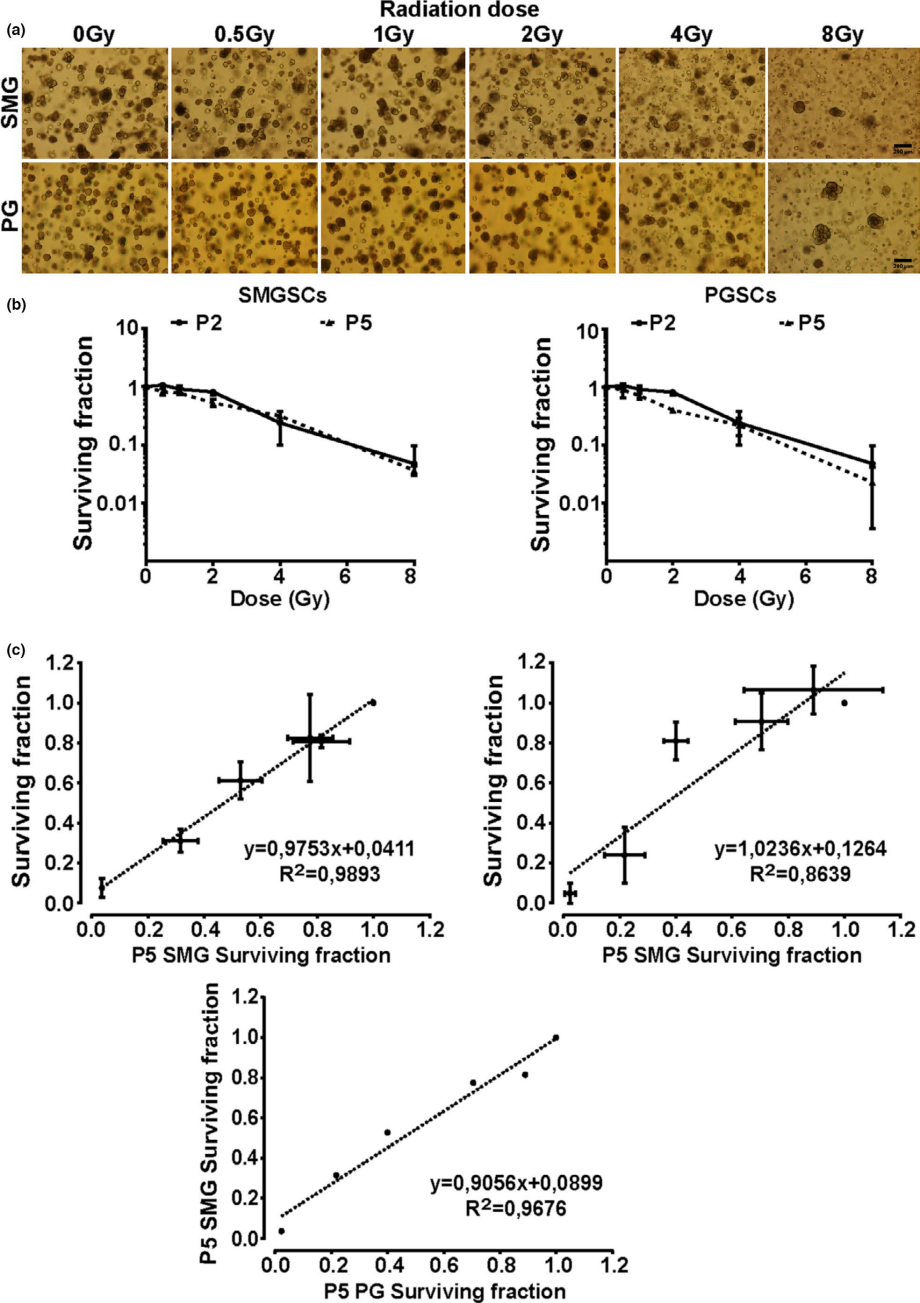
Radiation response of SMG and PG stem cells in vitro. (a) Representative images of the growth of non‐irradiated and irradiated PG and SMG stem cells. (b) Comparison of the radiation responses of different passages of PG and SMG stem cells to photon irradiation. (c) Scatter plots indicating a linear correlation between P2 and P5 surviving fraction either for the PG or the SMG and between the P5 surviving fraction of the PG and SMG. Error bars represent *SD*. Scale bars 50 μm. *N* = 4 biological repeats

Next we investigated the radiosensitivity of PG stem cells and compared them to the sensitivity of the SMG stem cells as described earlier (Nagle et al., 2016, 2018). Organoid‐derived single cells from P1 or P4 were (sham‐)irradiated and subsequently cultured as P2 and P5 in Matrigel with WRY medium. The surviving fraction of cells able to form new organoids was enumerated as a measure of surviving stem cells. Indeed, Figure 4a shows that with increasing radiation dose less organoids are formed. The radiation response of both PG and SMG stem cells did not significantly differ between different passages (Figure 4b,c) nor between the PG and SMG (Figure 4c, bottom). These data show that a potential difference in radiation sensitivity between the PG and SMG cannot be explained by differences in radiation sensitivity of their respective stem cells.

## 4 DISCUSSION

Although the PG is the most studied organ regarding radiation therapy of head and neck cancer, in vitro models resembling the in vivo situation for in depth mechanistic studies are lacking. Considering difficulties in obtaining human tissue, a murine model will help to elucidate mechanisms involved in PG regeneration. These mechanisms could be translated to the clinic in order to reduce hyposalivation‐related complaints and to improve quality of life of head and neck cancer patients. In the current study, we developed and tested an organoid model for the culture of murine PG stem cells. Based on previous work on SMG organoids as described by Nagle et al. (2016), 3D culture systems of SMG can yield “spheres” (cluster of cells, growing indefinitely) or “organoids,” which are structurally more defined, with branched and lobular structures, containing stem cells and other cell types of the tissue of origin (Maimets et al., 2016; Nagle et al., 2016; Nanduri et al., 2014). Here, we show for the first time the prolonged in vitro expansion of adult murine PG stem cells within this system and the potential to form lobular structure with differentiation capacities into the major salivary gland cell types, supporting two major characteristics defining stem cells and the definition of organoids. In addition, we highlighted the use of SG organoid models for the study of the response of different SG stem cells to irradiation. The use of this system leads to the observation that the irradiation response of stem cells derived from either the murine PG or SMG does not seem to differ. We also provide evidence for the important role of the canonical Wnt/beta‐catenin signaling in the maintenance of PG stem cells. Wnt signaling is broadly recognized as a key driver of the organoid formation from different adult epithelial cells, like the mammary gland (Jardé et al., 2016; Zeng & Nusse, 2010), the intestine (Farin, Van Es, & Clevers, 2012), and the SMG (Maimets et al., 2016). In murine SMG, an important role of Wnt signaling activation during the postnatal development and during tissue regeneration after ductal ligation has been demonstrated (Hai et al., 2010). In the case of SMG organoid‐derived stem cells, it has been reported that the activation of the Wnt pathway potentiates their self‐renewal potential and long‐term expansion in vitro (Maimets et al., 2016). Although SMG stem cells can be expanded without the addition of Wnt, the chemical inhibition of the Wnt/beta‐catenin signaling led to the abrogation of organoid formation. These observations culminated in the suggestion that stem cells derived from the SMG may be able to produce their own Wnt for their maintenance by means of a paracrine mechanism (Maimets et al., 2016). In contrast, we show here that PG stem cells need external Wnt signaling stimulation, since the generation of primary organoids is not possible without medium devoid of Wnt3A and R‐Spondin. Since for stem cell therapy it is important to obtain the patient's own (autologous) stem cells, biopsies will have to be acquired before radiotherapy. Taking into account that these biopsies will be rather small, the number of stem cells necessary for successful transplantation needs to be expanded. Therefore, it is crucial to study the role of Wnt signaling and other pathways involved in the maintenance and expansion of both PG and SMG stem cells and organoids.

For this, it is also necessary to further characterize the specific stem cells. Our approach toward this characterization by analyzing the potential enrichment of the stem cell population within the PG, using CD24/CD29 expression, demonstrated another difference with the SMG. CD24^high^/CD29^high^ cells clearly showed enhancement of organoid formation in the SMG (Nanduri et al., 2014). It was possible to differentiate subsets of CD24/CD29 populations similar to the SMG; however, none of these PG subpopulations led to an enhancement of the self‐renewal capacity. Notably, the study of the self‐renewal potential of the CD24/CD29 subpopulations of the SMG was done without the addition of Wnt, although gene expression analysis of the CD24^++^/CD29^++^ P0 versus P10 showed an upregulation in the expression of genes related with the Wnt pathway (Nanduri et al., 2014). These might explain the interglandular differences since paracrine Wnt production might benefit the stem cell potential of the CD24^++^/CD29^++^ population in the SMG. In this study, it was not possible to distinguish in the PG if external addition of Wnt stimulation agents were necessary for stem cell expansion. Therefore, characterization of expression of other known stem cell markers needs to be further explored to eventually assess the quality of the to be transplanted cell population for stem cell therapies.

It has been proposed that the radiosensitivity of the PG and SMG differ (Vissink et al., 2010, 2015). Moreover, long‐term assessment of rat SG function after clinically relevant fractionated radiation doses showed that the potential to regenerate was larger in the PG compared to the SMG (Coppes et al., 2002). However, our data suggest that the respective stem cell populations do not differ in radiosensitivity in vitro. Therefore, the differences in radiosensitivity may not be due to radiation‐induced stem cell survival. First of all, we determined the irradiation response of PG and SMG stem cells under the effect of Wnt activation. Importantly, a radioprotective effect of Wnt to irradiation damage of different tissues has been reported. Pretreatment with R‐Spondin, a secreted protein that acts via the amplification of the Wnt pathway, ameliorated in a dose‐dependent manner the development of mucositis induced by radiation in mice (Zhao et al., 2009). Moreover, administration of parathyroid hormone reduced radiation damage on osteoblasts, through the activation of the Wnt pathway leading to the suppression of cell death and enhanced DNA repair (Chandra et al., 2015). Transient overexpression of the Wnt1 prevented hyposalivation in irradiated mice, by inhibiting apoptosis (Hai et al., 2012). However, after comparison of our data with the SMG response to irradiation reported earlier by our group but without the addition of Wnt (Nagle et al., 2016), we did not see an enhancement of the survival fraction of SMG stem cells under Wnt activation. The PG response of stem cells to irradiation did not show discrepancy with the case of the SMG even though there is a Wnt signaling dependency of the PG cells. The lack of the observation of Wnt protection in our system might be related with the time frame of treatment (Hai et al., 2012) that was used, since the cells were under Wnt signaling for 2 hrs only pre‐irradiation. This might not be sufficient time to activate the pathway in order to lead to the inhibition of apoptosis. Indeed, several other mechanisms than DNA damage‐induced SG stem cell response have been shown to at least in part be responsible for (early) salivary gland function loss (Konings, Coppes, & Vissink, 2005). Early apoptosis of acinar cells has been suggested to participate in the early response of SG to irradiation (Kartachova et al., 2006). In animal models however, this was not convincingly demonstrated (Konings et al., 2005), as it was suggested that early radiation‐induced dysfunction was due to membrane damage‐related signaling effects (Coppes, Meter, Latumalea, Roffel, & Kampinga, 2005). Interestingly, microvascular injury may play a role in radiation‐induced SG function loss (Mizrachi et al., 2016). Possibly, this is modulated by Wnt stimulation. Vascularization of SG organoids, such as been developed for kidney organoids (Homan et al., 2019), may shed some light on these differences in mechanism in SG response to radiation. The co‐culture of vasculature and PG organoids will increase the physiological relevance of the mechanistic studies and relevance for potential future clinical use.

In conclusion, we have developed a murine PG organoid model by which unlimited expansion of multipotent stem cells can be achieved. Moreover, the PG and SMG depict similar in vitro stem cell radiosensitivity. Further exploration of SG organoid models should be used to investigate the mechanism of radiation response of SG and other normal tissue in vitro and help to develop a stem cell therapy for patients that are treated for head and neck cancer for the improvement of their quality of life.

## AUTHOR CONTRIBUTION

Paola Serrano Martinez: Data curation; Formal analysis; Funding acquisition; Investigation; Methodology; Visualization; Writing‐original draft. Davide Cinat: Data curation; Formal analysis; Investigation; Visualization; Writing‐review & editing. Peter van Luijk: Conceptualization; Investigation; Writing‐review & editing. Mirjam Baanstra: Investigation; Methodology. Gerald de Haan: Conceptualization; Supervision; Writing‐review & editing. Sarah Pringle: Conceptualization; Investigation; Methodology; Supervision; Writing‐review & editing. Robert Coppes: Conceptualization; Funding acquisition; Supervision; Visualization; Writing‐review & editing.

## Supporting information

Fig S1

Fig S2
