# Supplementary figures and images for: Mouse parotid salivary gland organoids for the in vitro study of stem cell radiation response
Source: Oral Dis. 2020 Jun 29;27(1):52–63. doi: 10.1111/odi.13475 (PMC7818507; doi:10.1111/odi.13475)

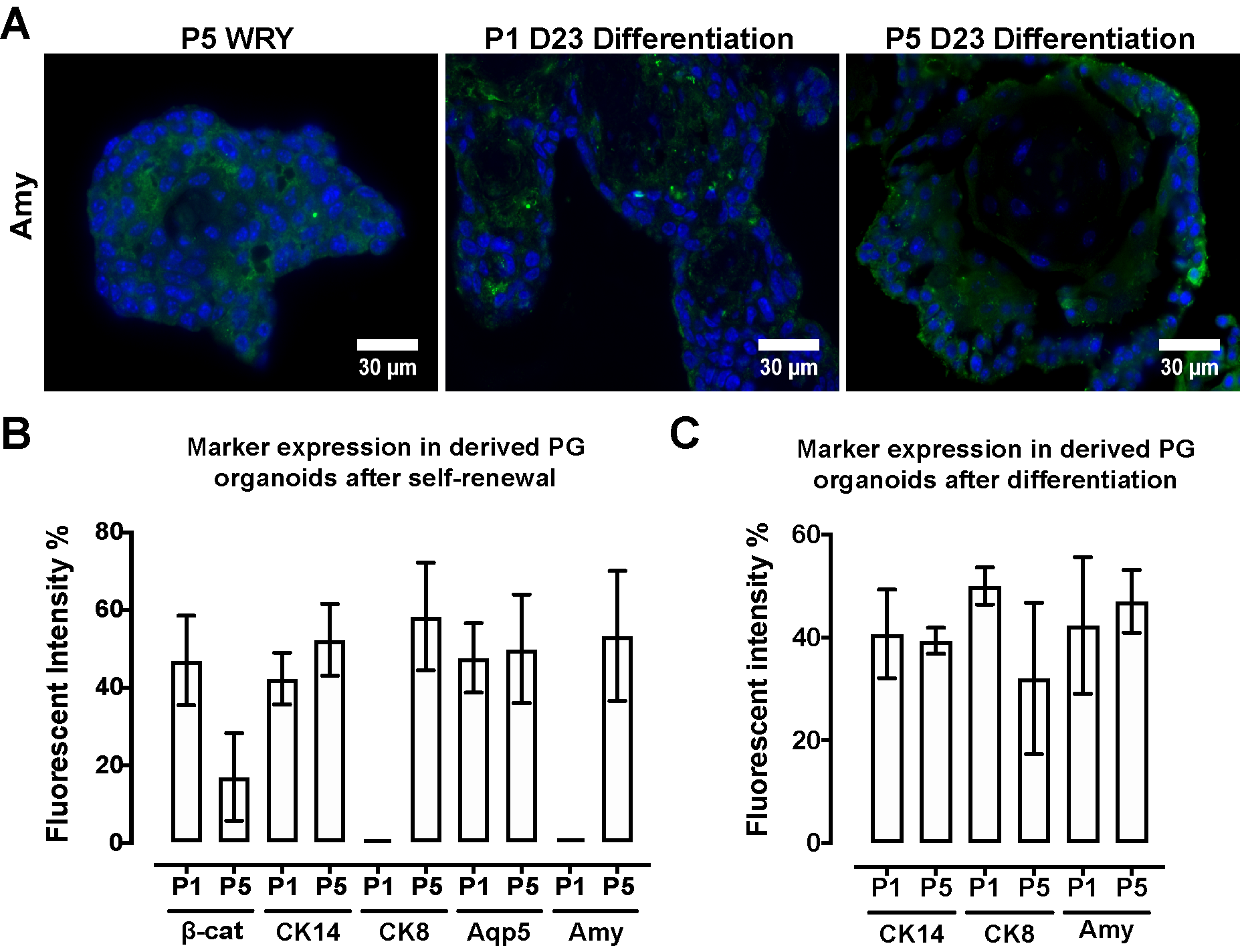

Supplement: Supplementary file 1 — Fig S1 [file ODI-27-52-s001.tif]

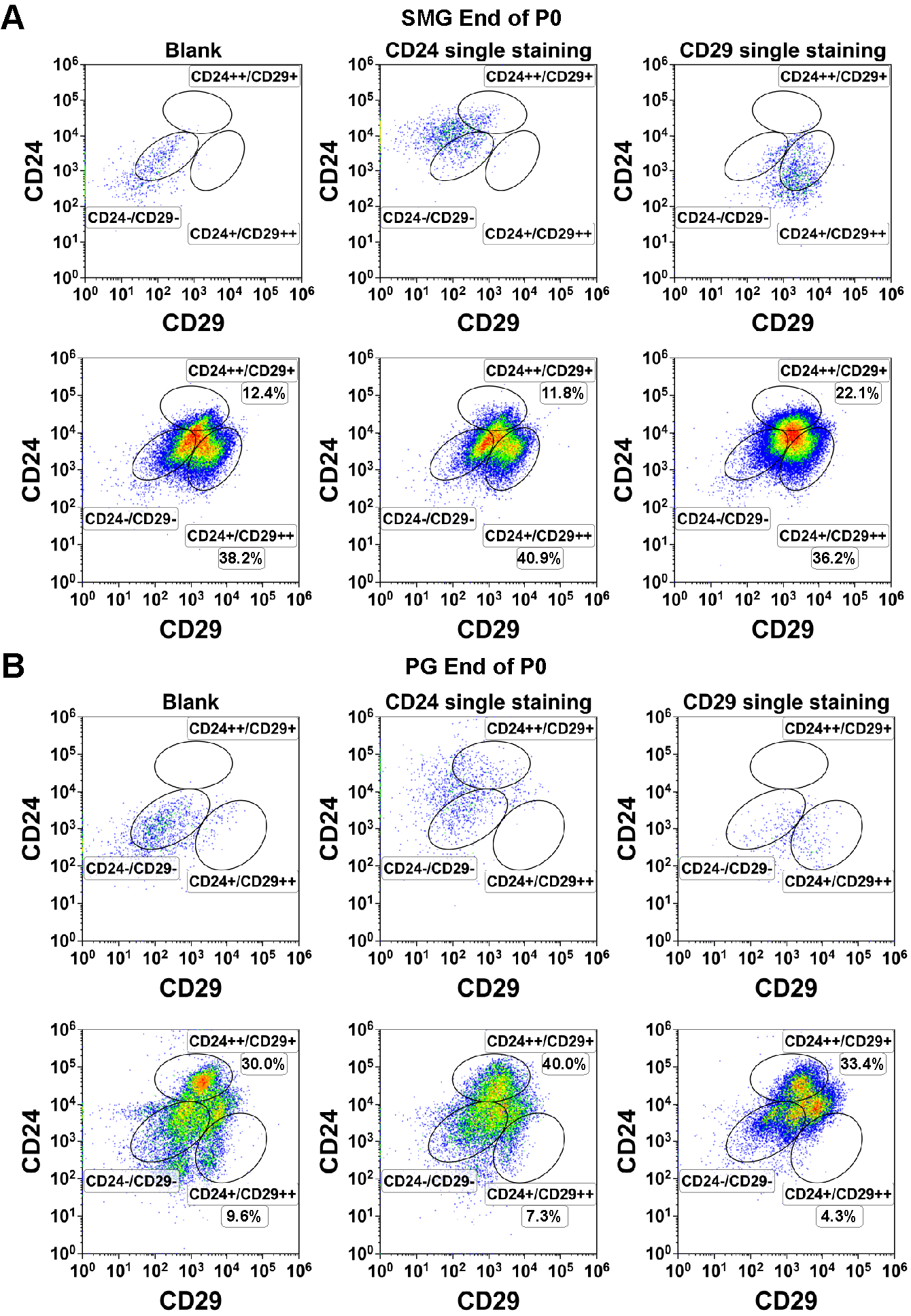

Supplement: Supplementary file 2 — Fig S2 [file ODI-27-52-s002.tif]
